# Assessment of Barriers to Dental Care Among Children in Saudi Arabia Using Levesque’s Framework: A Cross-Sectional Study

**DOI:** 10.3390/healthcare14152264

**Published:** 2026-07-24

**Authors:** Alaa A. Alkhateeb, Maram Alwadi, Shazia Khan, Haya Alayadi, Shekha Bin Muaily, Wafa Alshaibani

**Affiliations:** Dental Health Department, College of Applied Medical Sciences, King Saud University, P.O. Box 10219, Riyadh 11433, Saudi Arabia

**Keywords:** dental care, health services accessibility, child, oral health, parents, Saudi Arabia

## Abstract

**Background/Objectives:** Access to dental care is a multilevel challenge shaped by healthcare systems, individual characteristics, and broader social determinants. This study aimed to identify parent-reported barriers to dental care among a sample of children in Saudi Arabia using Levesque’s Conceptual Framework for Access to Healthcare. **Methods:** A cross-sectional survey was conducted between March and April 2023 among parents of children aged 1–18 years. The 44-item survey assessed sociodemographic, oral health, and dental care utilization, organized using Levesque’s access dimensions. Difficulty accessing needed dental care within the past 12 months served as the main outcome. The chi-square test was used to examine bivariate associations between potential barriers and difficulty accessing needed dental care. Multivariable logistic regression was used to identify factors independently associated with difficulty accessing needed dental care after adjustment for the other variables. **Results:** This study included 351 parents. The mean age of children was 10.7 ± 4.7 years, and 45.9% were male. One-third of parents reported difficulty accessing needed dental care for their children. The adjusted regression model identified fair/poor oral health (aOR = 13.39, 95% CI: 6.19–28.97), general health condition (aOR = 2.31, 95% CI: 1.11–4.82), and the absence of a nearby dental clinic (aOR = 2.08, 95% CI: 1.11–3.90) as factors independently associated with difficulty accessing dental care. **Conclusions:** Applying Levesque’s framework highlighted the multidimensional nature of barriers to accessing dental care for the study population. These findings provide preliminary evidence on parent-reported access barriers and may inform larger, representative studies and future policies to improve equitable pediatric oral healthcare in alignment with Saudi Vision 2030.

## 1. Introduction

Oral health is an essential aspect of overall health and well-being. Poor oral health in children can lead to pain, difficulties with eating and speaking, school absenteeism, and reduced self-esteem, consequently impacting their quality of life [1,2]. Dental caries remains one of the most common chronic diseases among children worldwide. A systematic review and meta-analysis estimated the global prevalence of untreated caries in primary teeth to be approximately 46.2% [3]. In Saudi Arabia, recent national data indicate high prevalence rates of dental caries among children, with an average of 75.4% in primary teeth and 67.7% in permanent teeth [4]. These findings highlight the continued need to strengthen preventive measures and improve access to dental care. In addition, the persistence of this high disease burden indicates that the availability of dental services alone may not ensure that children receive timely preventive and therapeutic care.

Access to dental care is a multidimensional concept that extends beyond the mere availability of services and the organization of healthcare systems [5,6,7,8,9]. It encompasses individuals’ ability to perceive their need for care, seek services, physically reach healthcare facilities, afford care, and ultimately receive appropriate treatment within the context of their family circumstances, community resources, cultural norms, and broader social determinants. Access should therefore be distinguished from service utilization. A child may live near a dental clinic but still experience difficulty obtaining care because of treatment costs, limited operating hours, transportation challenges, dental fear, lack of awareness of available services, or the inability of a parent to take time away from work. In addition, a dental visit does not necessarily indicate that access needs have been adequately met, particularly when care is delayed until pain develops or when treatment is incomplete. Access to dental care for children is also shaped by the central role of parents and caregivers. Children generally depend on adults to recognize oral health problems, decide whether professional care is necessary, arrange appointments, provide transportation, pay for treatment, and support adherence to professional recommendations. Consequently, barriers may arise from interactions among the child’s clinical needs, parental knowledge and resources, family circumstances, and the organization of dental services. Understanding these interactions is particularly important because difficulties at any stage of the care-seeking process may contribute to delayed treatment, symptom-driven attendance, or unmet dental care needs.

Over the past four decades, several frameworks have been developed to better understand healthcare access dynamics [5,6,7,8,9]. The most recent and comprehensive model is Levesque’s Conceptual Framework for Healthcare Access, published in 2013 [10]. This framework conceptualizes access as the interface between the characteristics of healthcare services and those of the population. It identifies five dimensions of accessibility: approachability, acceptability, availability and accommodation, affordability, and appropriateness, and five corresponding user abilities: ability to perceive, seek, reach, pay, and engage. The model offers a nuanced understanding of how individuals interact with health systems and is particularly valuable for identifying barriers and facilitators across multiple levels [10]. This framework is particularly relevant to pediatric dental care because it captures both service-related factors and the abilities and resources of parents or caregivers acting on behalf of their children. It also enables barriers to be examined as interconnected rather than as isolated financial, geographic, or behavioral factors.

Globally, evidence shows that children from disadvantaged backgrounds due to income, parental education, or rural residence experience disproportionate barriers to accessing dental care [11,12]. In Saudi Arabia, similar disparities persist despite the availability of public dental care and national oral health initiatives. Prior studies consistently report a range of barriers, including poor oral health literacy, financial difficulties, transportation challenges, and dentist-related concerns such as trust and availability [13,14]. Many families rely on symptom-driven visits, with pain being the primary reason for seeking care, while preventive visits remain limited [15,16,17]. Socioeconomic factors such as parents’ education and household income significantly influence care-seeking behaviours and regular dental attendance [14,15,16,17]. Geographic disparities further exacerbate access difficulties, particularly in underserved and remote areas with limited proximity to dental facilities [18]. Additionally, systemic issues, such as the lack of integration of dental care into general medical care, contribute to the underutilization of preventive dental care for children [19,20]. Collectively, these factors may contribute to inequalities in oral health, as children who experience difficulty accessing care may be more likely to receive treatment only after pain or other symptoms develop.

Although some recent Saudi studies have adopted theoretical models such as Andersen’s Behavioral Model to examine barriers and facilitators of dental care utilization [13], the existing research remains limited in scope. Several studies primarily target specific age groups [14] and are confined to single cities such as Al-Madinah [15], Dammam [16], or Jeddah [20]. Moreover, existing studies on barriers to dental care access among children in Saudi Arabia have not utilized structured, multidimensional frameworks to analyze access. Thus, this cross-sectional study aimed to assess parent-reported barriers to dental care among a sample of children in Saudi Arabia using Levesque’s Conceptual Framework for Access to Healthcare [10]. By examining both structural and individual-level factors, the study sought to provide an initial understanding of dental care access within the surveyed sample and to generate evidence that may inform larger, more representative studies. Such research may ultimately support the development of policies and services aimed at improving equitable access to pediatric oral healthcare in alignment with the broader objectives of Saudi Vision 2030.

## 2. Materials and Methods

### 2.1. Study Design and Study Population

This was a cross-sectional online survey study. STROBE (Strengthening the Reporting of Observational Studies in Epidemiology) checklist is provided as Supplementary File S1. This study was designed and coordinated at King Saud University, Riyadh, Saudi Arabia. Study participants were parents and caregivers of children aged 1 to 18 years old living in Saudi Arabia. Inclusion criteria were being a parent or legal caregiver of at least one child aged 1–18 years and currently residing in Saudi Arabia. Individuals who were unable to read Arabic or who did not provide informed consent were excluded. Parents could complete the survey for more than one child if multiple children met the eligibility criteria. For this study, we defined children as individuals aged 1–18 years, reflecting the age range for which parents typically act as primary decision-makers for dental care and in alignment with the Saudi Ministry of Health definition of pediatric patients. A non-probability convenience sample was recruited using online dissemination of the survey link. We included all complete responses in our study. The study was conducted from 11 March to 30 April 2023. This study was approved by the institutional review board at King Saud University, protocol no. E-23-7611. All procedures were conducted in accordance with the Declaration of Helsinki. Participation was voluntary, and electronic informed consent was obtained from all parents or caregivers before survey completion.

### 2.2. Survey

We designed a 44-item survey completed by participating parents on behalf of their children. The survey included questions about children’s sociodemographic characteristics (age, gender, geographical location, dental insurance, birth order, and household income), dental health factors (oral health status, current or history of oral pain or discomfort, previous dental visits, and oral hygiene practices (e.g., brushing, flossing)). The survey was developed by a multidisciplinary team of dental public health and pediatric dentistry experts. The questionnaire was developed de novo in Arabic, drawing on Levesque’s framework and prior Saudi and international literature on barriers to dental care. Content validity was assessed qualitatively through expert review, and the survey was refined based on their feedback. A small group of parents (n = 5) completed a pretest to evaluate clarity and comprehension, and minor wording changes were made accordingly. We designed the online survey using Google Forms and distributed it through social media platforms (e.g., X, WhatsApp). The full survey (original Arabic version and a translated English version) is provided as Supplementary File S2. Due to the open online dissemination and the absence of a predefined sampling frame, the total number of individuals who viewed the survey invitation and the overall response rate could not be determined.

### 2.3. Outcome Measure

Our outcome measure was difficulty accessing needed dental care in the past 12 months. The outcome was operationalized as a binary outcome from parents’ answers to the question: In the past 12 months, was there a time when your child needed dental care but was not able to get it (yes, no)?

### 2.4. Barriers to Needed Dental Care Mapped Using Levesque’s Conceptual Framework for Access to Healthcare

We mapped potential barriers to needed dental care using the five interrelated Levesque domains and mirrored user abilities. The first domain is approachability/ability to perceive care needs, which included perceived child oral health status; frequency of dental visits; age at first dental visit; time since last dental visit; dentist type; reason for the most recent dental visit; frequency of tooth or gum pain. The second domain is the acceptability/ability to seek, which included chronic health conditions and special needs status. The third domain is the availability and accommodation/ability to reach domain, which included the availability of a dental clinic near the home and the type of the nearest dental clinic. Fourth, the affordability/ability to pay domain included private dental insurance coverage and family income. Fifth, the appropriateness/ability to engage domain included the presence of unmet dental care needs in the prior 12 months, types of unmet dental care need, parent perceived barriers to obtaining needed dental care (lack of insurance; inability to afford treatment cost; transportation difficulties; clinic far from home; public clinics open only during regular working hours; parents unable to take time off work; fear of the dentist/dental setting; belief that the needed dental care was not urgent), school absenteeism to dental pain, difficulty eating due to dental pain, emergency-room visits due to dental pain, and home oral-hygiene practices.

### 2.5. Data Analysis

Descriptive statistics for the study population were presented using means and standard deviations (SD) for normally distributed quantitative variables and frequencies and percentages for categorical variables. For hypothesis testing, first, we used the chi-square test to assess the bivariate associations between potential barriers and difficulty accessing dental care. Second, we fitted a multivariable logistic regression model with difficulty accessing needed dental care as the dependent variable and child age, gender, father’s education, mother’s education, perceived oral health status, presence of a general health condition, availability of a nearby dental clinic, private dental insurance, and family income as predictors. We did not include special needs status as a predictor, because although it was mapped to the Levesque framework, only 5 participants were reported to have special needs, which produced an unstable estimate. Because of conceptual overlap with the outcome, we also excluded dental visit regularity, inability to afford treatment, lack of insurance as a reported barrier, missed school, emergency room visits, and difficulty eating from the regression model. Adjusted odds ratios (aOR) with 95% confidence intervals (CI) were reported. Although a formal a priori sample size calculation was not conducted, a sample of 351 parents was considered adequate for the descriptive and exploratory aims of the study and was comparable to or larger than samples used in previous studies assessing barriers to dental care access among children. Statistical significance was set at 0.05 for all statistical analyses, and all tests were two-sided. Data were analyzed using RStudio (version 2026.6.0.242).

## 3. Results

### 3.1. Sociodemographic Characteristics of the Study Population

A total of 351 participating parents completed the study survey (Table 1). The mean age of children was 10.7 ± 4.7 years, and 45.9% were male. Regarding residential region, 81.8% lived in the central part of Saudi Arabia, 9.7% lived in the west region, 4.6% lived in the east region, 2.8% lived in the north region, and 1.1% lived in the south region. Around 88.0% of children lived with both parents. In terms of the highest parental education, 10.0% of fathers and 3.7% of mothers had middle school education or lower, 29.9% of fathers and 17.4% of mothers had a high school education, and 60.1% of fathers and 78.9% of mothers had completed college education or higher.

### 3.2. Description of Child’s Dental Health-Related Factors Grouped by Levesque’s Framework Dimensions

#### 3.2.1. Approachability/Ability to Perceive

Approximately 85% of parents rated their child’s oral health good, very good, or excellent (Table 1). In terms of dental care utilization, around two-thirds of children had irregular dental visits only when experiencing pain. The average age at which children had their first dental visit was 6.1 ± 3.6 years. Regarding the child’s latest visit to the dental clinic, around two-thirds of children had visited a dental clinic within the past year. Pain and routine check-ups were the most commonly reported reasons for the last dental visit. Around 29.9% of parents reported that their child frequently complained of pain in the teeth or gums.

#### 3.2.2. Acceptability/Ability to Seek

Around 87.7% of children were reported to have no general health conditions, while 12.3% had at least one health condition (Table 1). Only 1.4% of children were reported as having special needs.

#### 3.2.3. Availability & Accommodation/Ability to Reach

In terms of dental care access, 80.9% reported having a dental clinic near their home (Table 1). In terms of the type of the nearest dental clinic, 64.7% reported a private dental clinic, and 20.8% reported a public dental clinic. Around 2.0% of parents reported inability to seek needed dental care for children due to transportation difficulties, and 1.7% reported the clinic being far from home.

#### 3.2.4. Affordability/Ability to Pay

In terms of family income, 34.5% of participating families reported earning 15,000 SAR or more per month (Table 1). More than two-thirds of children in this study did not have private dental health insurance. Among parents reporting unmet dental care needs, 15.5% reported lack of dental insurance and 25.0% reported inability to afford treatment as barriers.

#### 3.2.5. Appropriateness/Ability to Engage

Around one third of participating parents reported that their child was unable to obtain needed dental care in the last year (Table 1). Among these 116 children with unmet dental care needs, 42.2% needed dental fillings, 25.0% needed preventive care, and 14.7% needed extraction or surgical procedures. In terms of the barrier of not obtaining the needed dental treatment, 25% reported inability to afford the treatment cost, 19.8% reported fear of the dentist or dental setting, and 15.5% reported lack of dental insurance.

In terms of the impact of dental pain on children’s daily life activities, 14.0% of parents reported that their child had missed school because of tooth pain, 30.2% of parents reported that their child had difficulty eating because of tooth pain, and 10.3% of parents reported that their child had visited the emergency room because of tooth pain.

### 3.3. Outcome Measure

Of the participating parents, 33.0% reported difficulty obtaining needed dental care for their children.

### 3.4. Barriers to Dental Care Mapped by Levesque’s Framework Dimensions

#### 3.4.1. Sociodemographic Factors

There was no difference in age or gender between children who had difficulty accessing needed dental care and those who did not have difficulty (*p* = 0.098, *p* = 0.45, respectively) (Table 2). Father’s education was significantly associated with difficulty accessing needed dental care. Fathers of children with difficulty were more likely to have a middle school education or lower (with difficulty 12.9% vs. without difficulty 8.5%, *p* = 0.043), and less likely to have a college education (with difficulty 55.2% vs. without difficulty 62.6%, *p* = 0.043).

#### 3.4.2. Approachability/Ability to Perceive

Parents of children with difficulty accessing needed dental care reported significantly poorer child oral health status compared to those without difficulty (with difficulty 6.9% vs. without difficulty 0.9%, *p*< 0.001) (Table 2). This association remained significant after adjusting for other predictors (aOR = 13.39, 95% CI 6.19–28.97, *p* < 0.001) (Table 3). In terms of frequency of dental care visits, children with difficulty accessing dental care were significantly more likely to report irregular dental visits (with difficulty 75.0% vs. without difficulty 55.3%, *p* = 0.005) (Table 2).

There was no difference in age at first dental visit for children with and without difficulty accessing dental care (6.21± 3.40 years vs. 5.98 ± 3.73 years, *p* = 0.57). Similarly, no significant differences were observed in the reported time since the last dental visit or the type of dentist (*p* = 0.10, *p* = 0.19, respectively). The majority of children in both groups reported visiting pediatric dentists (with difficulty 54.3% vs. without difficulty 48.1%), followed by general dentists (with difficulty 39.7% vs. without difficulty 40.0%).

In terms of the reason of the last dental visit, children with difficulty accessing dental care were more likely to visit the dental clinic due to pain compared to those without difficulty (with difficulty 69.0% vs. without difficulty 44.7%, *p* < 0.001), less likely to visit for preventive services (with difficulty 15.5% vs. without difficulty 23.8%, *p* < 0.001), and less likely to visit for routine check-ups (with difficulty 15.5% vs. without difficulty 31.5%, *p* < 0.001). Children with difficulty were significantly more likely to frequently complain about tooth or gum pain compared to those without difficulty (with difficulty 56.9% vs. without difficulty 16.6%, *p* < 0.001).

#### 3.4.3. Acceptability/Ability to Seek

Children with difficulty accessing dental care were significantly more likely to have a general health condition compared to those without difficulty (with difficulty 19.8% vs. without difficulty 8.5%, *p* = 0.004). This association remained significant after adjusting for other predictors (aOR = 2.31, 95% CI 1.11–4.82, *p* = 0.026) (Table 3). Children with difficulty accessing dental care were significantly more likely to have special needs (with difficulty 3.4% vs. without difficulty 0.4%, *p*= 0.042) (Table 2).

#### 3.4.4. Availability and Accommodation/Ability to Reach

Children with difficulty accessing dental care were less likely to report having a nearby dental clinic compared to those without difficulty (with difficulty 73.3% vs. without difficulty 84.7%, *p*= 0.033). This association remained significant in the adjusted models. The absence of a nearby dental clinic was associated with higher odds of difficulty accessing dental care (aOR = 2.08, 95% CI: 1.11–3.90; *p* = 0.023) (Table 3). In terms of the type of nearby dental clinic, a lower proportion of parents of children with difficulty accessing dental care reported a nearby private dental clinic compared to those without difficulty (with difficulty 52.6% vs. without difficulty 70.6%, *p* = 0.003), and a higher proportion reported a nearby public dental clinic (with difficulty 27.6% vs. without difficulty 17.4%, *p*= 0.003) (Table 2).

#### 3.4.5. Affordability/Ability to Pay

There were no significant differences in terms of private dental insurance coverage between children with and without difficulty accessing dental care (with difficulty 19.8% vs. without difficulty 24.7%, *p* = 0.08). Similarly, family income distribution did not differ significantly between groups (*p* = 0.90).

#### 3.4.6. Appropriateness/Ability to Engage

Parents of children with difficulty accessing dental care were more likely to have missed school due to dental pain (with difficulty 31.9% vs. without difficulty 5.1%, *p* < 0.001) and to have visited the emergency room for dental pain (with difficulty 22.4% vs. without difficulty 4.3%, *p* < 0.001) (Table 2). Children with difficulty accessing dental care were also more likely to report difficulty eating due to dental pain (with difficulty 56.0% vs. without difficulty 17.4%, *p* < 0.001). No significant differences were observed in tooth brushing frequency between groups (*p* = 0.13) or in flossing frequency (*p* = 0.58).

## 4. Discussion

This cross-sectional study used Levesque’s Conceptual Framework for Access to Healthcare to assess barriers to dental care among a sample of children in Saudi Arabia [10]. Consistent with Cu et al.’s observations, the application of Levesque’s framework in this study revealed that its dimensions are not always discrete [21]. Despite this overlap, the framework enhanced our understanding of access as a dynamic interaction between patients and dental care providers rather than a linear pathway, offering a coherent structure to interpret the barriers experienced by Saudi children. Around one-third of participating parents in this study reported that their children had difficulty accessing needed dental care in the last year. In addition, the study identified 18 barriers to accessing needed dental care, which are grouped by the five dimensions and abilities of Levesque’s framework (A conceptual mapping of the identified barriers onto Levesque’s framework is provided in Supplementary Figure S1).

First, approachability refers to the characteristics of dental services that make them visible, identifiable, and reachable to families. Its corresponding population ability is the ability to perceive the need for dental care, which, in this study, largely depends on parents and caregivers [10]. This study identified four significant barriers associated with parents’ ability to perceive the need for dental care, which was significantly associated with difficulty accessing needed dental care. The first barrier was parents’ perception of their child’s oral health. Parents of children who experienced difficulty accessing needed dental care reported significantly poorer child oral health compared to those who did not experience difficulty accessing dental care. This is consistent with another observational study of barriers to oral health of children in Saudi Arabia, which showed that children who faced barriers to oral health were more likely to have poor oral health [14]. This highlights how caregivers’ awareness of oral health problems is central to recognizing the need for care yet may not necessarily translate into effective service use when care options are not visible or approachable. Similarly, the frequency and reasons for dental visits further illustrated this pattern. Children with difficulty accessing care in this study were significantly more likely to have irregular dental visits that occurred only when experiencing pain and to visit primarily because of pain. They were also less likely to seek preventive care or routine check-ups. This finding is consistent with a study on dental care utilization among schoolchildren in Saudi Arabia, which reported that more than two-thirds of children with untreated dental caries had never visited a dentist before [13]. Furthermore, children with difficulty accessing needed dental care reported higher complaints of tooth or gum pain than their counterparts. This was also consistent with findings of the study of the barriers to oral health of children in Saudi Arabia, which showed that more than half of the children who were facing barriers to oral health had constant oral pain [14]. Collectively, these findings reveal a reactive rather than preventive pattern of care-seeking, indicating that caregivers’ perception of need is often triggered by acute symptoms rather than preventive awareness. Within Levesque’s approachability dimension, this suggests that while caregivers may recognize oral health problems, systemic and informational barriers, such as limited outreach, unclear pathways to services, and insufficient promotion of preventive dentistry, hinder their ability to act upon this recognition. These barriers can be enhanced by increasing the visibility and outreach of dental care services for children, particularly preventive ones, which may strengthen the approachability of services and encourage earlier and more regular utilization. Dental outreach programs are identified as effective facilitators for enhancing approachability.

Second, acceptability refers to the social and cultural factors that influence whether individuals are willing to seek and engage with dental services [10]. This dimension emphasizes the consistency between patients’ expectations and the characteristics of the care provided. The corresponding ability, the ability to seek, reflects the parents’ motivation and willingness to pursue dental care for the child. In this study, three significant factors were associated with this dimension. Children with chronic health conditions were more likely to experience difficulty accessing dental care compared to those without such conditions. Similarly, children with special needs were also more likely to report difficulty accessing care. This is consistent with several studies exploring the barriers to dental care for children with disability [22,23,24,25]. Additionally, fear of the dentist and dental settings was more common among those reporting difficulty accessing needed care. This is consistent with a study examining the impact of dental fear on oral health–related behaviors, which found that children with higher levels of dental fear reported irregular and less frequent dental care utilization [24]. Collectively, these findings highlight how both medical vulnerability and psychosocial factors influence the acceptability of dental care services. The presence of chronic health conditions or special needs may amplify parents’ concerns about their child’s comfort, safety, and the provider’s ability to manage complex cases. Similarly, dental fear, whether stemming from previous negative experiences or a negative perception of dental settings, may discourage families from seeking timely care even when services are available. Within Levesque’s framework, these results underscore the importance of strengthening caregivers’ trust and emotional comfort with dental providers through culturally sensitive communication, behavior management strategies, and inclusive clinical environments.

Third, availability and accommodation relate to the organizational and geographical aspects of dental services that determine whether care can be reached conveniently [10]. The corresponding ability, the ability to reach, reflects the parents’ capacity to physically access and utilize available services. In this study, we identified several barriers to dental care that are related to dental care availability and the ability of parents to access care. Parents of children with difficulty accessing needed dental care were less likely to report having a nearby dental clinic, and less likely to identify private clinics as the closest option. This finding is consistent with previous studies demonstrating that greater travel distance and limited proximity to dental clinics are significant barriers to dental care utilization among children and families [25,26]. These studies from different contexts have shown that longer travel distances reduce the likelihood of routine dental visits and that patients often bypass nearby clinics due to perceived differences in quality or service type. Moreover, practical barriers such as transportation difficulties, the clinic being far from home, and public clinics operating only during regular working hours were more commonly reported barriers in our study. In addition, some caregivers reported the inability to take time off work as a constraint to attending dental appointments. Similarly, other studies have identified transportation challenges, inconvenient clinic hours, and competing work responsibilities as key obstacles to accessing dental services. Research from various settings has shown that parents who face limited transportation options or inflexible work schedules are less likely to bring their children for routine dental visits, underscoring the impact of logistical and time-related constraints on dental care utilization [27,28,29,30]. Collectively, these findings emphasize how geographic proximity, clinic type, and service scheduling can shape families’ ability to reach dental care. Even when services are available, their concentration in specific locations or limited operating hours can render them effectively inaccessible for working parents. Within Levesque’s framework, these barriers underscore the interplay between service supply and family circumstances. To address barriers related to availability and accommodation, expanding community-based dental services, integrating flexible appointment systems, and increasing the availability of after-hours or school-based clinics could substantially improve the accommodation and accessibility of care for children.

Fourth, affordability reflects the economic capacity of individuals and households to pay for services and the relationship between the cost of care and their ability to engage with it [10]. The corresponding ability, the ability to pay, captures the caregiver’s financial resources and willingness to allocate them toward dental care. In this study, affordability emerged as a commonly reported barrier among parents whose children had unmet dental care needs. Among these parents, inability to afford treatment and lack of dental insurance were among the most frequently reported barriers. This is consistent with several national and international studies that have identified the cost of care as one of the most frequently reported barriers to accessing dental services [13,14,29,30]. These findings underscore that despite the availability of public dental services in Saudi Arabia, financial barriers persist, particularly for families relying on private clinics or specialized services that may not be covered under available public dental services. Within Levesque’s framework, limited affordability restricts caregivers’ ability to act upon recognized needs even when other dimensions, such as approachability or availability, are met. Addressing affordability, therefore, requires not only expanding insurance coverage for preventive and restorative services but also increasing public awareness of free or low-cost options within the primary healthcare system to ensure equitable access to oral health care.

An apparent discrepancy in this study warrants closer examination. Parent-reported barriers, including lack of dental insurance and inability to afford treatment costs, were commonly reported among parents whose children had unmet dental care needs. However, actual private dental insurance coverage and family income were not significantly associated with difficulty accessing needed dental care. This pattern may reflect that perceived affordability, benefit coverage, out-of-pocket costs, and structural insurance/income status capture related but distinct aspects of financial access. Having private insurance or a higher income does not guarantee that a family perceives dental treatment as affordable, particularly if copayments, uncovered services, or specialist fees are involved, whereas not having insurance does not necessarily translate into a reported access barrier if free or subsidized public services are used instead. Parent-reported barrier items may also be more proximally linked to the specific unmet-need episode being reported and are conceptually close to the outcome, whereas insurance and income are more distal, structural indicators.

Finally, appropriateness refers to the fit between the services provided and the patient’s health needs, encompassing the quality, continuity, and timeliness of care [10]. The corresponding ability, the ability to engage, reflects parents’ and children’s capacity to participate in and benefit from care once it is reached. In this study, several indicators related to the consequences of unmet dental needs were significantly associated with difficulty accessing care. Children with difficulty accessing needed dental care were far more likely to have missed school due to dental pain, to have visited the emergency room for dental pain, and to have experienced difficulty eating because of dental pain. These findings align with previous observational studies from Saudi Arabia demonstrating that dental caries can significantly affect children’s daily lives, contributing to school absences, emergency dental visits, and impaired eating and well-being [14,30]. These findings indicate that difficulty accessing dental care co-occurred with indicators of functional burden, including school absence, emergency room visits, and difficulty eating due to dental pain. Because of the cross-sectional design, the temporal relationship between access difficulty and these outcomes cannot be established. Within Levesque’s framework, such outcomes indicate deficiencies not only in service availability but also in the appropriateness and responsiveness of care delivery. When children experience pain severe enough to disrupt eating or schooling, it reflects gaps in preventive engagement and follow-up continuity. These findings underscore the need for integrated oral health services within school and primary care systems, emphasizing preventive and child-centred continuous care to reduce the functional and educational impacts of dental pain among children.

This study has several limitations. First, its cross-sectional design limits causal inference. The associations between identified barriers and difficulty in accessing dental care cannot be interpreted as causal or directional relationships. Second, the sample size (N = 351), while adequate for detecting moderate associations, remains relatively small and may limit the power to identify subtle differences across subgroups or less common barriers. In addition, the study used an online, non-probability convenience sample recruited through social media. Because the number of individuals who viewed the survey invitation could not be determined, a response rate could not be calculated, and self-selection bias cannot be excluded. Moreover, although participants were recruited from all major regions of Saudi Arabia, most respondents resided in the central region. The findings may therefore not capture regional differences in the organization, availability, and accessibility of dental services and should be interpreted as reflecting the experiences of the surveyed parents rather than as nationally representative estimates for all children in Saudi Arabia. Information on the specific type of residential setting, such as village, small city, or large city, was also not collected, limiting the assessment of geographic gradients in access beyond broad regional classifications. Third, data were based on parent-reported variables, which may be subject to recall bias, particularly regarding children’s oral health behaviors and service utilization. Fourth, the survey was pretested with five parents to assess clarity and comprehension; however, formal validity and reliability assessments were not conducted. Consequently, the measurement properties of the instrument could not be fully evaluated. Fifth, not all dimensions of Levesque’s health care access framework were fully captured in this study, including aspects related to trust and communication with providers, travel time, and waiting time. Moreover, assigning study variables to the framework’s conceptual dimensions involved some overlap and may have introduced interpretive subjectivity. Sixth, because multiple bivariate comparisons were conducted, the possibility of type I error cannot be excluded; therefore, the unadjusted bivariate *p*-values should be interpreted cautiously, and the bivariate findings should be regarded as exploratory. Finally, certain contextual factors, such as provider attitudes and health-system policies, were not assessed but may further influence access. Future studies should use larger, geographically representative samples, clinically verified oral-health measures, formally validated questionnaires, and mixed-methods or longitudinal designs to provide a more comprehensive understanding of barriers to pediatric dental care.

## 5. Conclusions

Applying Levesque’s framework provided a structured, multidimensional view of parent-reported barriers to dental care among a sample of children in Saudi Arabia. The findings revealed interconnected challenges across individual, social, and system levels, suggesting a potential need to enhance the visibility, inclusivity, and responsiveness of dental services. These exploratory findings may inform future studies and support policy discussions aimed at reducing financial barriers, improving the geographic availability of child-friendly dental services, and promoting earlier, preventive care, particularly for children with chronic conditions and special needs. Addressing these dimensions collectively may support progress toward more equitable and sustainable oral health outcomes for Saudi children. Larger studies with more geographically representative samples are needed to confirm the findings of this study.

## Figures and Tables

**Table 1:** Description of study participants grouped by Levesque’s Framework Dimensions (N = 351).

Variable	N (%)
**Sociodemographic characteristics**	
**Child age in years** (mean ± SD)	10.7 ± 4.7
**Child gender**	
Male	161 (45.9%)
Female	190 (54.1%)
**Residential region**	
Middle	287 (81.8%)
East	16 (4.6%)
West	34 (9.7%)
North	10 (2.8%)
South	4 (1.1%)
**Child living arrangement by parental marital status**	
Child lives with both parents	309 (88.0%)
Child lives with mother	29 (8.3%)
Child lives with father	7 (2.0%)
Other	6 (1.7%)
**Father’s highest education**	
Middle school or lower	35 (10.0%)
High school	105 (29.9%)
College education or higher	211 (60.1%)
**Mother’s highest education**	
Middle school or lower	13 (3.7%)
High school	61 (17.4%)
College education or higher	277 (78.9%)
**Approachability/Ability to Perceive**	
**Child oral health status**	
Excellent	43 (12.3%)
Very good	159 (45.3%)
Good	95 (27.1%)
Fair	44 (12.5%)
Poor	10 (2.8%)
**Frequency of dental visit:**	
Regularly every 6 months	46 (13.1%)
Regularly every year	35 (10.0%)
Irregularly—only when experiencing pain	217 (61.8%)
Never visits	49 (14.0%)
I don’t know	4 (1.1%)
**Age at first dental visit** (mean ± SD)**:**	6.1 ± 3.6 years
**Time of last dental visit:**	
3 months or less	105 (29.9%)
6 months or less	40 (11.4%)
More than 6 months but less than a year	79 (22.5%)
2 years or more	79 (22.5%)
I don’t know	48 (13.7%)
**Type of dentist:**	
General Dentist	140 (39.9%)
Pediatric Dentist	176 (50.1%)
I don’t know	35 (10.0%)
**Reason for last dental visit**	
Routine check-up	92 (26.2%)
Preventive services	74 (21.1%)
Pain in teeth or gums	185 (52.7%)
**Child frequently complains about pain in teeth or gum**	
Yes	105 (29.9%)
No	229 (65.2%)
I don’t know	17 (4.8%)
**Acceptability/Ability to Seek**	
Have general health conditions	
Yes	43 (12.3%)
No	308 (87.7%)
Special needs	
Yes	5 (1.4%)
No	346 (98.6%)
**Availability & Accommodation/Ability to Reach**	
**Dental clinic near your home:**	
Yes	284 (80.9%)
No	35 (10.0%)
Maybe	19 (5.4%)
I don’t know	13 (3.7%)
**Type of clinic near home:**	
Public	73 (20.8%)
Private	227 (64.7%)
I don’t know	51 (14.5%)
**Affordability/Ability to Pay**	
**Private dental health insurance**	
Yes	81 (23.1%)
No	258 (73.5%)
I don’t know	12 (3.4%)
**Family income**	
Less than 6999 SAR	42 (12.0%)
7000–13,999 SAR	117 (33.3%)
15,000–21,999 SAR	72 (20.5%)
22,000 SAR and above	49 (14.0%)
I don’t know	17 (4.8%)
I prefer not to disclose	54 (15.4%)
**Appropriateness/Ability to Engage**	
**Could not get needed dental care in the last 12 months**	
Yes	116 (33.0%)
No	235 (67.0%)
**Types of unmet dental treatment need (N = 116)**	
Restorative	49 (42.2%)
Extraction/surgery	17 (14.7%)
Preventive	29 (25.0%)
Others	19 (16.4%)
**Barriers to obtaining needed dental care (N = 116)**	
Lack of dental insurance	18 (15.5%)
Unable to afford the treatment cost	29 (25.0%)
Transportation difficulties	4 (3.4%)
Clinic is far from home	4 (3.4%)
Public dental clinics operate only during work hours	10 (8.6%)
Unable to take time off or leave from work	5 (4.3%)
Fear of the dentist and dental settings	23 (19.8%)
I believe the needed dental care is not urgent	5 (4.3%)
Other	15 (12.9%)
**During the past 12 months, did your child miss school due to tooth pain?**	
Yes	49 (14.0%)
No	295 (84.0%)
I don’t know	7 (2.0%)
**During the past 12 months, did your child have difficulty eating due to tooth pain?**	
Yes	106 (30.2%)
No	222 (63.2%)
I don’t know	23 (6.6%)
**Has your child ever visited the emergency room due to tooth pain since birth?**	
Yes	36 (10.3%)
No	311 (88.6%)
I don’t know	4 (1.1%)
**Tooth brushing frequency**	
Once a day	166 (47.3%)
Twice or more a day	103 (29.3%)
Once or twice a week	61 (17.4%)
Never	9 (2.6%)
I don’t know	12 (3.4%)
**Does the child receive help or supervision from a parent while brushing at home?**	
Yes	164 (46.7%)
No	179 (51.0%)
I don’t know	8 (2.3%)
**Toothbrush type:**	
Soft	164 (46.7%)
Medium	134 (38.2%)
Hard	3 (0.9%)
Electric	19 (5.4%)
I don’t know	2 (0.6%)
**Toothpaste type:**	
With fluoride	186 (53.0%)
Without fluoride	75 (21.4%)
For sensitive teeth	34 (9.7%)
Whitening toothpaste	10 (2.8%)
Doesn’t use toothpaste	8 (2.3%)
I don’t know	38 (10.8%)
**Flossing frequency**	
Once a day	24 (6.8%)
Twice or more a day	9 (2.6%)
Once or twice a week	21 (6.0%)
Never	258 (73.5%)
I don’t know	39 (11.1%)

Note: “Types of unmet dental treatment need” and “Barriers to obtaining needed dental care” were conditional items intended only for parents who reported unmet dental care needs in the past 12 months (n = 116); percentages for these two items were calculated using n = 116 as the denominator. All other percentages were calculated using the full sample (N = 351).

**Table 2:** Association between participants’ characteristics and difficulty accessing dental care (N = 351).

Independent Variable	Difficulty Accessing Dental Care		*p*-Value *
	Yes N = 116	No N = 235	
**Sociodemographic characteristics**			
**Age categories**			0.098
1–5 years	10 (8.6%)	33 (14.0%)	
6–10 years	53 (45.7%)	84 (35.7%)	
11–15 years	31 (26.7%)	83 (35.3%)	
16–18 years	20 (17.2%)	32 (13.6%)	
Missing	2 (1.7%)	3 (1.3%)	
**Child gender**			0.45
Male	57 (49.1%)	104 (44.3%)	
Female	59 (50.9%)	131 (55.7%)	
**Residential region**			0.47
Middle	97 (83.6%)	189 (80.4%)	
East	4 (3.4%)	9 (3.8%)	
West	7 (6.0%)	29 (12.3%)	
North	5 (4.3%)	5 (2.1%)	
South	2 (1.7%)	3 (1.3%)	
**Father’s highest education**			**0.043**
Middle school or lower	15 (12.9%)	20 (8.5%)	
High school	37 (31.9%)	68 (28.9%)	
College education or higher	64 (55.2%)	147 (62.6%)	
**Mother’s highest education**			0.13
Middle school or lower	7 (6.0%)	6 (2.6%)	
High school	24 (20.7%)	37 (15.7%)	
College education or higher	85 (73.3%)	192 (81.7%)	
**Approachability/Ability to Perceive**			
**Child oral health status**			**<0.001**
Excellent	6 (5.2%)	37 (15.7%)	
Very good	30 (25.2%)	129 (54.9%)	
Good	37 (31.9%)	58 (24.7%)	
Fair	35 (30.2%)	9 (3.8%)	
Poor	8 (6.9%)	2 (0.9%)	
**Frequency of dental visit:**			**0.005**
Regularly every 6 months	8 (6.9%)	38 (16.2%)	
Regularly every year	8 (6.9%)	27 (11.5%)	
Irregularly—only when experiencing pain	87 (75.0%)	130 (55.3%)	
Never visits	13 (11.2%)	36 (15.3%)	
I don’t know	0.0 (0.0%)	4 (1.7%)	
**Age at first dental visit** (mean ± SD):	6.21 ± 3.40	5.98 ± 3.73	0.57
**Time of last dental visit:**			0.10
3 months or less	35 (30.2%)	70 (29.8%)	
6 months or less	20 (17.2%)	20 (8.5%)	
More than 6 months but less than a year	24 (20.7%)	55 (23.4%)	
2 years or more	26 (22.4%)	53 (22.6%)	
I don’t know	11 (9.5%)	37 (15.7%)	
**Type of dentist:**			0.19
General Dentist	46 (39.7%)	94 (40.0%)	
Pediatric Dentist	63 (54.3%)	113 (48.1%)	
I don’t know	7 (6.0%)	28 (11.9%)	
**Reason for last dental visit**			**<0.001**
Routine check-up	18 (15.5%)	74 (31.5%)	
Preventive services	18 (15.5%)	56 (23.8%)	
Pain in teeth or gums	80 (69.0%)	105 (44.7%)	
**Child frequently complains about pain in teeth or gum**			**<0.001**
Yes	66 (56.9%)	39 (16.6%)	
No	40 (34.5%)	189 (80.4%)	
I do not know	10 (8.6%)	7 (3.0%)	
**Acceptability/Ability to Seek**			
**Child has general health conditions**			**0.004**
Yes	23 (19.8%)	20 (8.5%)	
No	93 (80.2%)	215 (91.5%)	
**Child has special needs**			**0.042**
Yes	4 (3.4%)	1 (0.4%)	
No	112 (96.6%)	234 (99.6%)	
**Availability & Accommodation/Ability to Reach**			
**Dental clinic nearby**			**0.033**
Yes	85 (73.3%)	199 (84.7%)	
No	16 (13.8%)	19 (8.1%)	
Maybe	7 (6.0%)	12 (5.1%)	
I do not know	8 (6.9%)	5 (2.1%)	
**Type of nearby dental clinic**			**0.003**
Public dental clinic	32 (27.6%)	41 (17.4%)	
Private dental clinic	61 (52.6%)	166 (70.6%)	
Missing	23 (19.8%)	28 (11.9%)	
**Affordability/Ability to Pay**			
**Child has private dental insurance**			0.08
Yes	23 (19.8%)	58 (24.7%)	
No	92 (79.3%)	166 (70.6%)	
I do not know	1 (0.9%)	11 (4.7%)	
**Family income**			0.90
Less than 6999 SAR	16 (13.8%)	26 (11.1%)	
7000–13,999 SAR	39 (33.6%)	78 (33.2%)	
15,000–21,999 SAR	20 (17.2%)	52 (22.1%)	
22,000 SAR and above	16 (13.8%)	33 (14.0%)	
I don’t know	6 (5.2%)	11 (4.7%)	
I prefer not to disclose	19 (16.4%)	35 (14.9%)	
**Appropriateness/Ability to Engage**			
**Missed school due to dental pain**			**<0.001**
Yes	37 (31.9%)	12 (5.1%)	
No	77 (66.4%)	218 (92.8%)	
I do not know	2 (1.7%)	5 (2.1%)	
**Child visited emergency room due to dental pain**			**<0.001**
Yes	26 (22.4%)	10 (4.3%)	
No	87 (75.0%)	224 (95.3%)	
I do not know	3 (2.6%)	1 (0.4%)	
**Child has difficulty eating due to dental pain**			**<0.001**
Yes	65 (56.0%)	41 (17.4%)	
No	48 (41.4%)	174 (74.0%)	
I do not know	3 (2.6%)	20 (8.5%)	
**Tooth brushing frequency**			0.13
Once a day	51 (44.0%)	115 (48.9%)	
Twice or more a day	30 (25.9%)	73 (31.1%)	
Once or twice a week	29 (25.0%)	32 (13.6%)	
Never	3 (2.6%)	6 (2.6%)	
I don’t know	3 (2.6%)	9 (3.8%)	
**Flossing frequency**			0.58
Once a day	9 (7.8%)	15 (6.4%)	
Twice or more a day	5 (4.3%)	4 (1.7%)	
Once or twice a week	8 (6.9%)	13 (5.5%)	
Never	81 (69.8%)	177 (75.3%)	
I don’t know	13 (11.2%)	26 (11.1%)	

* Chi-square test. Bolded *p*-value < 0.05.

**Table 3:** Multivariable logistic regression analysis of factors associated with difficulty accessing needed dental care (N = 351).

Predictor	aOR	95% CI	*p*-Value
Child age, per one-year increase	1.01	0.95–1.07	0.77
Female sex (ref: male)	0.83	0.50–1.38	0.47
Father’s education: high school or lower (ref: college education or higher)	1.03	0.43–2.46	0.94
Mother’s education: high school or lower (ref: college education or higher)	1.86	0.47–7.29	0.37
Fair/poor perceived oral health (ref: good/very good/excellent)	13.39	6.19–28.97	**<0.001**
General health condition present (ref: none)	2.31	1.11–4.82	**0.026**
No nearby dental clinic (ref: nearby clinic available)	2.08	1.11–3.90	**0.023**
No private dental insurance (ref: private dental insurance)	1.14	0.62–2.10	0.68
Insurance status unknown (ref: private dental insurance)	0.12	0.01–1.22	0.07
Family income ≥15,000 SAR (ref: <15,000 SAR)	1.10	0.61–1.96	0.76

Estimates were obtained from a single multivariable logistic regression model including all variables shown. The dependent variable was parent-reported difficulty accessing needed dental care during the past 12 months. Bolded *p*-value < 0.05.

## Data Availability

The datasets generated and analyzed during the current study are not publicly available due to containing potentially identifying or sensitive patient information, but are available from the corresponding author on reasonable request.

## Supplementary Materials

The following supporting information can be downloaded at: https://www.mdpi.com/article/10.3390/healthcare14152264/s1.
