# Supplementary material for: Assessment of Barriers to Dental Care Among Children in Saudi Arabia Using Levesque’s Framework: A Cross-Sectional Study
Source: Healthcare (Basel). 2026 Jul 24;14(15):2264. doi: 10.3390/healthcare14152264 (PMC13464404; doi:10.3390/healthcare14152264)
Supplement: Supplementary file 1 [file healthcare-14-02264-s001.zip › healthcare-4368448-supplementary.pdf]

Supplementary File S1.

STROBE Statement—Checklist of items that should be included in reports of ***cross-sectional studies***

	Item No	Recommendation	Page No
**Title and abstract**	1	(*a*) Indicate the study's design with a commonly used term in the title or the abstract	1
		(*b*) Provide in the abstract an informative and balanced summary of what was done and what was found	1
**Introduction**			
Background/rationale	2	Explain the scientific background and rationale for the investigation being reported	2
Objectives	3	State specific objectives, including any prespecified hypotheses	2
**Methods**			
Study design	4	Present key elements of study design early in the paper	3
Setting	5	Describe the setting, locations, and relevant dates, including periods of recruitment, exposure, follow-up, and data collection	3-4
Participants	6	(*a*) Give the eligibility criteria, and the sources and methods of selection of participants	3-4
Variables	7	Clearly define all outcomes, exposures, predictors, potential confounders, and effect modifiers. Give diagnostic criteria, if applicable	4
Data sources/ measurement	8*	For each variable of interest, give sources of data and details of methods of assessment (measurement). Describe comparability of assessment methods if there is more than one group	*3-4*
Bias	9	Describe any efforts to address potential sources of bias	5
Study size	10	Explain how the study size was arrived at	3
Quantitative variables	11	Explain how quantitative variables were handled in the analyses. If applicable, describe which groupings were chosen and why	5
Statistical methods	12	(*a*) Describe all statistical methods, including those used to control for confounding	5
		(*b*) Describe any methods used to examine subgroups and interactions	NA
		(*c*) Explain how missing data were addressed	5
		(*d*) If applicable, describe analytical methods taking account of sampling strategy	NA
		(*e*) Describe any sensitivity analyses	NA
**Results**			
Participants	13*	(a) Report numbers of individuals at each stage of study—eg numbers potentially eligible, examined for eligibility, confirmed eligible, included in the study, completing follow-up, and analysed	5
		(b) Give reasons for non-participation at each stage	NA
		(c) Consider use of a flow diagram	NA
Descriptive data	14*	(a) Give characteristics of study participants (eg demographic, clinical, social) and information on exposures and potential confounders	5
		(b) Indicate number of participants with missing data for each variable of interest	5-14
Outcome data	15*	Report numbers of outcome events or summary measures	5-14

Main results	16	(*a*) Give unadjusted estimates and, if applicable, confounder-adjusted estimates and their precision (eg, 95% confidence interval). Make clear which confounders were adjusted for and why they were included	5-14
		(*b*) Report category boundaries when continuous variables were categorized	NA
		(*c*) If relevant, consider translating estimates of relative risk into absolute risk for a meaningful time period	NA
Other analyses	17	Report other analyses done—eg analyses of subgroups and interactions, and sensitivity analyses	NA
**Discussion**			
Key results	18	Summarise key results with reference to study objectives	14-18
Limitations	19	Discuss limitations of the study, taking into account sources of potential bias or imprecision. Discuss both direction and magnitude of any potential bias	17-18
Interpretation	20	Give a cautious overall interpretation of results considering objectives, limitations, multiplicity of analyses, results from similar studies, and other relevant evidence	14-18
Generalisability	21	Discuss the generalisability (external validity) of the study results	14-18
**Other information**			
Funding	22	Give the source of funding and the role of the funders for the present study and, if applicable, for the original study on which the present article is based	18

Supplementary File S2. Original questionnaire (Arabic), as administered via Google Forms

عوامل ومحددات الوصول إلى خدمات رعاية الأسنان للأطفال والمراهقين في المملكة العربية السعودية (استبيان لأولياء الأمور)

رقم السؤال	السؤال	خيارات الإجابة
1	عمر الابن/الابنة (بالسنوات)	إدخال رقمي
2	جنس الابن/الابنة	ذكر / أنثى
3	منطقة السكن	قائمة تضم جميع مناطق/محافظات المملكة العربية السعودية
4	هل يوجد عيادة أسنان قريب من المنزل؟	نعم / لا / ربما / لا أعلم
5	إذا كانت إجابتك للسؤال السابق نعم، ما نوع العيادة؟	حكومية / خاصة
6	هل يعاني الابن/الابنة من مشاكل صحية؟	نعم / لا
7	إذا كانت إجابتك للسؤال السابق نعم، ماهي المشاكل الصحية التي يعاني منها الابن/الابنة؟	نص مفتوح
8	هل الابن/الابنة من ذوي الاحتياجات الخاصة؟	نعم / لا
9	عدد إخوة الابن/الابنة	1-3 / 4-6 / 7 وأكثر
10	ترتيب الابن/الابنة في العائلة	الأكبر / الأوسط / الأصغر
11	هل يوجد تأمين لخدمات طب الأسنان للابن/الابنة؟	نعم / لا / لا أعلم
12	كيف تقيم صحة فم وأسنان الابن/الابنة؟	ممتاز / جيد جداً / جيد / مقبول / ضعيف
13	هل يشتكي الابن/الابنة بشكل متكرر من ألم في الأسنان أو اللثة؟	نعم / لا / لا أعلم
14	متى يقوم الابن/الابنة بزيارة عيادة الأسنان؟	بشكل دوري كل 6 أشهر / بشكل دوري كل سنة / بشكل غير دوري – عند الإحساس بألم فقط / لا يزور إطلاقاً / لا أعلم
15	في أي عمر كانت أول زيارة للابن/الابنة لعيادة الأسنان؟	إدخال رقمي (بالسنوات)
16	ما نوع الطبيب الذي يزوره الابن/الابنة؟	طبيب أسنان عام / طبيب أسنان للأطفال / لا أعلم
17	آخر زيارة لعيادة الأسنان قبل:	3 أشهر أو أقل / 6 أشهر أو أقل / أكثر من 6 أشهر وأقل من سنة / سنتين وأكثر / لا أعلم
18	ماذا كان سبب زيارة الابن/الابنة لعيادة الأسنان؟	فحص دوري / تنظيف / بسبب ألم في أسنانه أو اللثة
19	آخر مرة شعر فيها الابن/الابنة بألم في أسنانه أو لثته قبل:	3 أشهر أو أقل / 6 أشهر أو أقل / سنة وأكثر / لم يشعر بألم في الأسنان أو اللثة من قبل / لا أعلم
20	خلال الـ 12 شهراً الماضية، هل احتاج الابن/الابنة لزيارة طبيب الأسنان ولم يستطع؟	نعم / لا [المتغير التابع الأساسي]
21	إذا كان الجواب نعم للسؤال السابق، ما نوع العلاج الذي احتاجه الابن/الابنة ولم يحصل عليه؟	حشوات أسنان / خلع / علاج وقائي / أخرى
22	إذا كان الجواب أخرى للسؤال السابق، اذكر نوع العلاج	نص مفتوح
23	ما الذي منع الابن/الابنة من الحصول على العلاج الذي احتاجه؟	عدم وجود تأمين / عدم القدرة على دفع مبلغ العلاج / صعوبة في المواصلات / العيادة بعيدة عن المنزل / اقتصار عمل المستشفيات الحكومية على الفترة الصباحية / عدم القدرة على أخذ إجازة أو استئذان من العمل / الخوف من طبيب الأسنان / أعتقد أن الأمر أو المشكلة غير طارئة / أخرى [اختيار واحد، مقصود عرضه فقط عند الإجابة بنعم على السؤال 20 – انظر الملاحظة أدناه]
24	خلال الـ 12 شهراً الماضية، هل تغيب الابن/الابنة عن المدرسة بسبب ألم الأسنان؟	نعم / لا / لا أعلم
25	خلال الـ 12 شهراً الماضية، هل عانى الابن/الابنة من صعوبة في الأكل بسبب ألم الأسنان؟	نعم / لا / لا أعلم

26	هل سبق وزار الابن/الابنة الطوارئ بسبب ألم الأسنان منذ ولادته؟	نعم / لا / لا أعلم
27	إذا كانت إجابتك للسؤال السابق نعم، كم مرة زار الابن/الابنة الطوارئ بسبب ألم الأسنان منذ ولادته؟	نص/رقم مفتوح
28	كم عدد مرات تفريش الابن/الابنة؟	مرة واحدة في اليوم / مرتين أو أكثر في اليوم / مرة أو مرتين خلال الأسبوع / إطلاقاً / لا أعلم
29	هل يتلقى الابن/الابنة مساعدة أو يتم الإشراف عليه من أحد الوالدين خلال التفريش في المنزل؟	نعم / لا / لا أعلم
30	نوع الفرشاة:	ناعمة / متوسطة / خشنة / إلكترونية / لا أعلم
31	نوع معجون الأسنان:	معجون بدون فلورايد / معجون بالفلورايد / معجون لأسنان الحساسة / معجون للتبييض / لا يستعمل معجون / لا أعلم
32	كم عدد مرات استعمال الخيط للابن/الابنة؟	مرة واحدة في اليوم / مرتين أو أكثر في اليوم / مرة أو مرتين خلال الأسبوع / إطلاقاً / لا أعلم
33	كم عدد مرات استعمال المسواك للابن/الابنة؟	مرة واحدة في اليوم / مرتين أو أكثر في اليوم / مرة أو مرتين خلال الأسبوع / إطلاقاً / لا أعلم
34	كم عدد مرات استعمال غسول الفم للابن/الابنة؟	مرة واحدة في اليوم / مرتين أو أكثر في اليوم / مرة أو مرتين خلال الأسبوع / إطلاقاً / لا أعلم
35	من يقوم بتعبئة الاستبيان؟	الأم / الأب / غير ذلك
36	الحالة الاجتماعية للوالدين:	متزوجين / منفصلين، يسكن عند الأم / منفصلين، يسكن عند الأب / أخرى
37	الجنسية:	نص مفتوح
38	مستوى التعليم (للأب):	أمّي / ابتدائي / متوسط / ثانوي / جامعي أو أعلى
39	مستوى التعليم (للأم):	أمّية / ابتدائي / متوسط / ثانوي / جامعي أو أعلى
40	الحالة الوظيفية للأب:	موظف / غير موظف
41	إذا كان الأب موظفاً، كم مدة ساعات العمل؟	6-8 ساعات / 9-12 ساعة / 12 ساعة وأكثر
42	الحالة الوظيفية للأم:	موظفة / غير موظفة
43	إذا كانت الأم موظفة، كم مدة ساعات العمل؟	6-8 ساعات / 9-12 ساعة / 12 ساعة وأكثر
44	مجموع دخل العائلة الشهري:	أقل من 7 آلاف ريال / 7-14 ألف ريال / 15-22 ألف ريال / 22 ألف ريال وأكثر / لا أعلم / لا أريد الإفصاح
45	هل ترغب في تقديم أي اقتراحات أو توصيات؟	نص مفتوح (اختياري)

English translation of the study questionnaire

Item #	Question (English translation)	Response options
1	Age of the child (in years)	Numeric entry
2	Gender of the child	Male / Female
3	Residential area	Dropdown list of all Saudi regions/governorates.
3a	If your residential area is not listed above, please specify	Open text
4	Is there a dental clinic near your home?	Yes / No / Maybe / I don't know
5	If your answer to the previous question was yes, what type of clinic?	Government / Private
6	Does your child suffer from any health problems?	Yes / No
7	If your answer to the previous question was yes, what health problems does your child have?	Open text
8	Is your child among people with special needs?	Yes / No
9	Number of your child's siblings	1–3 / 4–6 / 7 or more
10	Your child's birth order in the family	Oldest / Middle / Youngest
11	Is there dental insurance for your child?	Yes / No / I don't know
12	How would you rate your child's oral and dental health?	Excellent / Very good / Good / Fair / Poor
13	Does your child frequently complain of pain in the teeth or gums?	Yes / No / I don't know
14	When does your child visit the dental clinic?	Regularly every 6 months / Regularly every year / Irregularly – only when experiencing pain / Never visits / I don't know
15	At what age was your child's first visit to a dental clinic?	Numeric entry (years)
16	What type of dentist does your child visit?	General dentist / Pediatric dentist / I don't know
17	Your child's last visit to a dental clinic was:	3 months or less / 6 months or less / More than 6 months but less than a year / 2 years or more / I don't know
18	What was the reason for your child's last dental visit?	Routine check-up / Cleaning (preventive) / Because of pain in the teeth or gums
19	Your child last felt pain in the teeth or gums:	3 months or less / 6 months or less / A year or more / Has never felt tooth/gum pain before / I don't know
20	In the past 12 months, was there a time your child needed to visit a dentist but could not?	Yes / No [Primary outcome variable]
21	If yes to the previous question, what type of treatment did your child need but not receive?	Dental fillings / Extraction / Preventive treatment / Other
22	If "Other" to the previous question, please specify the type of treatment	Open text
23	What prevented your child from receiving the needed treatment?	Lack of insurance / Inability to afford treatment cost / Transportation difficulties / Clinic far from home / Government hospitals operate mornings only / Unable to take time off/leave from work / Fear of the dentist / Believed the matter was not urgent / Other [Single-select; intended to be shown only if Q20 = Yes – see note below]
24	In the past 12 months, did your child miss school because of tooth pain?	Yes / No / I don't know
25	In the past 12 months, did your child have difficulty eating because of tooth pain?	Yes / No / I don't know
26	Has your child ever visited the emergency room because of tooth pain since birth?	Yes / No / I don't know
27	If yes to the previous question, how many times has your child visited the emergency room for tooth pain since birth?	Open/numeric text

28	How many times does your child brush their teeth?	Once a day / Twice or more a day / Once or twice a week / Never / I don't know
29	Does your child receive help or supervision from a parent while brushing at home?	Yes / No / I don't know
30	Type of toothbrush	Soft / Medium / Hard / Electric / I don't know
31	Type of toothpaste	With fluoride / Without fluoride / For sensitive teeth / Whitening toothpaste / Doesn't use toothpaste / I don't know
32	How many times does your child use dental floss?	Once a day / Twice or more a day / Once or twice a week / Never / I don't know
33	How many times does your child use a miswak (traditional teeth-cleaning twig)?	Once a day / Twice or more a day / Once or twice a week / Never / I don't know
34	How many times does your child use mouthwash?	Once a day / Twice or more a day / Once or twice a week / Never / I don't know
35	Who is completing this questionnaire?	Mother / Father / Other
36	Parents' marital/social status	Married / Separated – child lives with mother / Separated – child lives with father / Other
37	Nationality	Open text
38	Father's highest education level	Illiterate / Primary / Middle school / Secondary (high school) / College or higher
39	Mother's highest education level	Illiterate / Primary / Middle school / Secondary (high school) / College or higher
40	Father's employment status	Employed / Not employed
41	If the father is employed, how many hours does he work?	6–8 hours / 9–12 hours / 12 hours or more
42	Mother's employment status	Employed / Not employed
43	If the mother is employed, how many hours does she work?	6–8 hours / 9–12 hours / 12 hours or more
44	Total monthly family income	Less than 7,000 SAR / 7,000–14,000 SAR / 15,000–22,000 SAR / 22,000 SAR or more / I don't know / Prefer not to disclose
45	Would you like to offer any suggestions or recommendations?	Open text (optional)

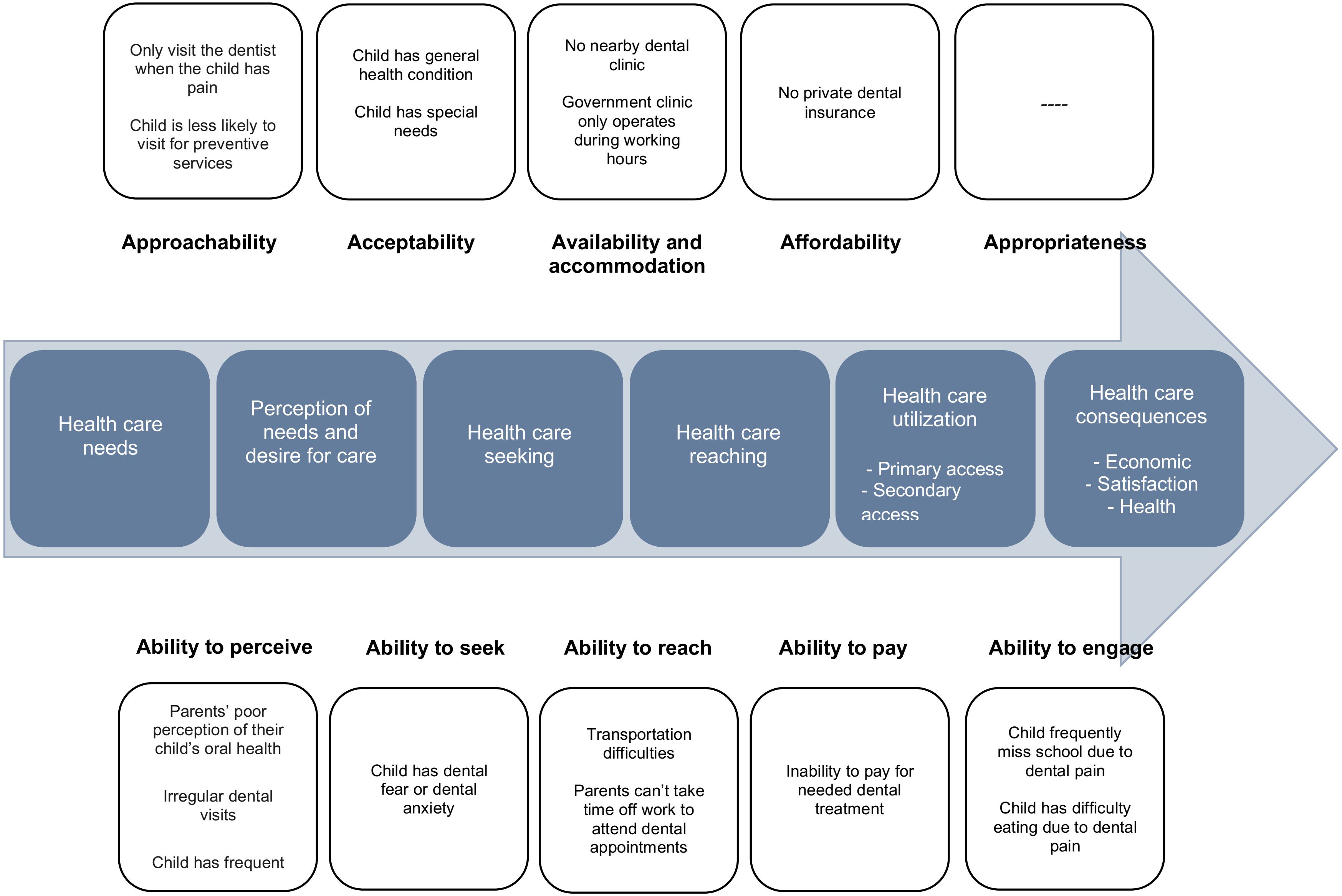

Supplementary Figure S1. Determinants of difficulty in accessing dental care for children mapped using Levesque's framework for access to health care.
